# The Effect of Alkali Treatment on Properties of Dopamine Modification of Bamboo Fiber/Polylactic Acid Composites

**DOI:** 10.3390/polym10040403

**Published:** 2018-04-04

**Authors:** Jianyong Lin, Zexun Yang, Xiaoxia Hu, Gonghua Hong, Shuangbao Zhang, Wei Song

**Affiliations:** Beijing Key Laboratory of Wood Science and Engineering, Beijing Forestry University, Beijing 100083, China; javanlin@bjfu.edu.cn (J.L.); zxyang@bjfu.edu.cn (Z.Y.); xiaoxiahu@bjfu.edu.cn (X.H.); honggonghua@bjfu.edu.cn (G.H.)

**Keywords:** bamboo fiber, polylactic acid, composites, dopamine modification, alkali treatment

## Abstract

In this study, a synergistic treatment including dopamine (DA) modification and alkali pretreatment on bamboo fiber (BF) was used as reinforcement in a polylactic acid (PLA) matrix to improve the mechanical and thermal properties of BF/PLA composites. The effects of the sodium hydroxide loading rate on the performance of mussel-inspired dopamine-modified bamboo fiber and the BF/PLA composites were evaluated using Fourier transform infrared spectroscopy (FTIR), X-ray diffraction (XRD), mechanical testing (examining flexural, tensile, and impact properties), differential scanning calorimetry (DSC), thermogravimetric analysis (TGA), and scanning electron microscopy (SEM). Analysis of the composites suggested that the optimal condition was treatment with a 4 wt % solution of NaOH and a 1 wt % concentration of dopamine. Compared with the untreated bamboo fiber/polylactic acid composites, the synergistic treatment improved the thermal properties and mechanical properties; flexural, tensile, and impact strengths increased by 16.1%, 34.4%, and 3.7%, respectively. It was further verified that appropriate alkali treatment was a promising approach in promoting the effect of dopamine-modified coating while maintaining the crystal structure of the cellulose.

## 1. Introduction

Many natural fiber polymer composites are being produced and applied. The composites are mainly used as reinforcement, and the thermoplastic polymer is used as the matrix. Natural fibers are beneficial for their low cost, low density, high strength, and high modulus [1]. Bamboo resources in China are abundant, and bamboo fiber is of interest to researchers for its outstanding mechanical properties. Its stiffness and strength are comparable to those of glass fiber [2,3]. Thus, composites with bamboo fiber-reinforcements are becoming popular because of their low cost, weather resistance, and biodegradability [4]. Polylactic acid (PLA) is an aliphatic polyester that has good biodegradable properties and biocompatibility. It is also widely used in plastic products, packaging, and medical implant materials. Many of the PLA properties (such as stiffness and tensile strength) are compared to those of polyethylene (PE), polypropylene (PP) and polyvinyl chloride (PVC). In addition, its thermal properties and mechanical properties are similar to those of polyethylene and polypropylene, which are considered an alternative to traditional petroleum-based products [5]. However, it is restricted by a high production cost, poor thermal stability, and brittleness. Therefore, the preparation of composite materials by mixing bamboo fiber with PLA can improve performance, reduce production cost, and remedy PLA’s defects while ensuring its original biodegradation performance [6,7].

However, bamboo fiber contains a large number of hydrophilic polar groups, and the interface compatibility between the hydrophilic and the hydrophobic polymer matrix is poor [8]. Hence, surface modification of bamboo fiber is a necessary step for fiber-reinforced polylactic acid composites. Research findings indicate that the chemical treatment is effective and stable, including alkali treatment, silane treatment, enzyme treatment, benzoylation treatment, and maleated coupling treatment [9,10,11]. Among various chemical treatments, alkali treatment has been the most widely used and effective method. For instance, Tokoro et al. observed a significant increase in the mechanical and thermal properties of bamboo fiber/PLA composites when the bamboo fiber was treated with sodium hydroxide in conjunction with mechanical processing [12]. Researchers have now begun to use alkali treatment as a pretreatment method to enhance the effectiveness of other chemical treatments. Asumani et al. found that alkali/silane-treated kenaf fiber showed better mechanical properties than either alkali-treated or silane-treated composites [13,14,15].

Recently, a mussel protein with super adhesion ability inspired by the marine mussel byssus gland is gradually applied in various fields. Studies have shown that at least five 3,4-dihydroxyphenyl alanine (DOPA)-containing adherent proteins are found in proteins secreted by marine mussel byssus gland [16,17]. Further studies have concluded that the catechol groups, the characteristic structure of the DOPA molecule, have a chemical function and hydrophilic diversity. Its phenol group can form strong hydrogen bonds with other polar polymers, providing the possibility of adhesion for mussel byssus protein. Dopamine is a catechol derivative of DOPA that is highly reactive, while it has the catechol group of DOPA and the amino functional group of lysine [18,19]. Dopamine can be used as a starting point of graft modification that can achieve universal infiltration and super adhesion to different substrates by forming chemical bonds with various kinds of matrix materials. It is an important basis to solve the interfacial compatibility between the polar, hydrophilic bamboo fiber and the non-polar, hydrophobic polymer. Meanwhile, dopamine can also serve as a secondary reflecting platform for the oxidative self-polygeneration of polydopamine (PDA) to firmly attach to the surface of a solid substrate and exhibit similar super-binding properties [20]. Hence, dopamine molecules show phenolic hydroxyl groups, amino groups close to the substrate, benzene rings, and hydrocarbon groups far away from the substrate during bamboo fiber interfacial treatment, exhibiting effective infiltration and reducing free hydroxyl groups from the surface of bamboo fiber, leading to reduction in the polarity of the bamboo fiber and thus improving the properties of the interface between bamboo fiber and polylactic acid [21]. Hong et al. had applied PDA to the interfacial modification of bamboo fiber/PBS composites, reporting obvious improvements in the mechanical and thermal properties when the concentration of dopamine is 1.0 mg/mL [22]. Zhou et al. introduced dopamine as a modifier for the surface treatment of ramie fiber and indicated that dopamine could be a green and novel surface modifier for natural fiber and that the control of interfacial crystallization could be an efficient way of enhancing the interface between the matrix and the fillers [23]. In previous research, PDA has been widely used to modify various fibers, but dopamine without polymerization has not been applied to fiber-reinforced polymer composites.

In this work, bamboo fiber was first treated with aqueous sodium hydroxide with different concentrations (4 wt %, 6 wt %, and 8 wt %, respectively) and then modified in aqueous dopamine with an immobilized concentration (1 wt %). Within this framework, the removal of impurities on the surface of bamboo fiber by alkali treatment greatly increases the effective contact area between the fiber and the matrix. In addition, the fibrous fibrils exposed in the fiber increase the number of reactive hydroxyl groups on the surface, and dopamine-modified coating is the process of hydrogen bonding between the phenolic hydroxyl groups or the amino groups of dopamine molecule and the hydroxyl groups of the bamboo fiber. Consequently, this study is expected to provide a comprehensive understanding of the behavior of this fiber during the processing of alkali treatment and dopamine modification.

## 2. Materials and Methods

### 2.1. Materials

The bamboo fiber (with a water content of 10%) of 40–60 mesh size and a length below 380 µm was purchased from Sentai Wood Plastic Composites Material Co., Ltd. (Huzhou, China). The polylactic acid (PLA), grade 4032 D, which was used as a polymer matrix, has a melting point of 160 °C with a density of 1.24 g/cm^3^. PLA was supplied by NatureWorks (Northford, CT, USA) Ind. Co., Ltd. Dopamine hydrochloride (98.5%) (DA) was purchased from Macklin Biochemical Co., Ltd. (Shanghai, China) and used directly. Other chemicals used in the study including sodium hydroxide and deionized water were purchased from Beijing Chemical Industry Group Co., Ltd. (Beijing, China).

### 2.2. Preparation of Alkali-Treated and DA-Modified Bamboo Fiber

DA modification: The bamboo fiber was thoroughly washed with tap water to remove any debris, dust, and other impurities and then dried in an air-dry oven (Marit, DHG-6030A, Wuxi, China) at 8 °C before treatment [24]. Next, the bamboo fiber was soaked in a dopamine hydrochloride solution with a fixed ratio, in which DA was 1 wt % dissolved in deionized water. Then the solution was stirred at 300 r/min for 8 h at atmospheric temperature (25 °C). Finally, before it was dried at 80 °C in an air-dry oven, the DA-modified bamboo fiber was washed with deionized water several times.

Alkali treatment: According to the typical process [25], the bamboo fiber was dipped in sodium hydroxide solution (with varying concentrations of 4%, 6%, and 8 wt % dissolved in deionized water), and the solution was then stirred at 300 r/min for 8 h in the water bath at 40 °C. The bamboo fiber was then washed with deionized water several times until the solution was presented to be neutral. After the desiccation of the alkali-treated bamboo fiber, these steps were repeated for dopamine modification.

Untreated, DA-modified, and alkali/DA co-treated BF were used to reinforce PLA, and the final composite materials were named as UB/PLA, DB/PLA, and ADB/PLA, respectively, as listed in Table 1.

### 2.3. Preparation of the Bamboo Fiber/PLA Composites

In the preparation of the composites in this experiment, the proportion of bamboo fiber was 40 wt %. The compounding of PLA with untreated and DA-modified BFs, with and without alkali-pretreatment, was accomplished using a co-rotating twin-screw extruder (KESUN KS20, Kunshan, China). The twin screw rotation speed was maintained at 100 rpm, and the temperature from the inlet to the head were set as follows: 165, 170, 175, 175, 165, and 160 °C. The mixtures were chopped until the diameter of the particles was 4 mm, and the particles were then hot-compression-molded (HAPCO, BY 602 × 2/2 150 T Testing Press, Suzhou, China) for 6 min at 170 °C and a pressure of 4 MPa. Before full-press, the sample was pre-heated for 3 min at 170 °C. Finally, the sample was cold-pressed for 10 min at 4 MPa and at room temperature via cold compression (CANGAO, CGYJ-100, Shijiazhuang, China).

### 2.4. Characterization

#### 2.4.1. Fourier Transform Infrared Spectroscopy (FTIR)

Untreated, DA-modified, and DA/alkali-treated BFs were subjected to FTIR analysis. Powder samples with a particle size less than 200 mesh were dispersed by potassium bromide (KBr) at a weight ratio of 1:100. The spectra were recorded by Vertex 70 v (BRUKER, Karlsruhe, Germany) obtained in transmission mode at a scanning range from 4000 cm^−1^ to 400^−1^ cm^−1^ with a resolution of 4 cm^−1^. Each group was measured three times to eliminate any error.

#### 2.4.2. X-ray Diffraction Procedure and Analysis (XRD)

Samples were analyzed by a D8 Advanced X-ray diffractometer (BRUKER, AXS, Germany) using $C_{u}K_{\alpha }$ radiation (*λ* = 0.154060 nm), a radiation tube voltage of 40 kV and a current of 40 mA, and the scanning range performed is 2θ = 5°–40° in the steps of $5^{{}^{\circ}}/\min$. Cellulose crystallinity index (*I_XRD_*) was calculated by the following equation [26,27]:

$$I_{XRD}\left(\%\right)\;=\;\frac{I_{002}-I_{101}}{I_{002}}\times 100$$

where *I*_002_ is the maximum intensity of the 002 lattice diffraction plane at an angle of between 22° and 23°, and *I*_101_ is the intensity diffraction at an angle close to 17° representing amorphous areas in the cellulosic fiber.

#### 2.4.3. Mechanical Characterization

The tensile test was measured using MODEL AG-IS (SHIMADZU, Kyoto, Japan) based on ASTM D638-03. The specimens had dimensions of 165 × 20 × 4 mm. The middle test part width was 13 mm. Eight specimens were measured with a crosshead speed of 5 mm/min.

The flexural test was measured using MODEL AG-IS (SHIMADZU, Kyoto, Japan) based on ASTM D790-03. The specimens had dimensions of 80 × 10 × 4 mm. Eight specimens were measured with a crosshead speed of 2 mm/min.

The impact test was measured using MODEL CREE-1002C (KERUI, XJJ5, Dongguan, China) based on ASTM D256-03. The specimens had dimensions of 80 × 10 × 4 mm. Eight specimens were measured with a crosshead speed of 2.9 m/s, with a 1 J hammer. These tests were carried out at room temperature.

#### 2.4.4. Thermogravimetric (TG) Analysis

The thermal stability of the composites samples was tested with a TA Instrument Q5000 apparatus (New Castle, DE, USA). The analysis was carried out by heating the samples from 40 to 600 °C at a rate of 10 °C/min under an N_2_ gas flow of 50 mL/min.

#### 2.4.5. Differential Scanning Calorimetry (DSC)

The thermal and crystalline properties of the composites samples were tested with a differential scanning calorimeter (Netzsch DSC-204, Bavaria, Germany). The samples were analyzed within a temperature range of 40–200 °C at a nitrogen flow rate of 60 mL/min at the same heating and cooling rates of 10 °C/min in three scans: heating, cooling, and heating. The crystallinity, *X_DSC_*, was calculated by the following equation [28,29,30]:

$$X_{DSC}\;=\;\frac{\Delta Hm}{f\cdot \Delta H_{m}^{0}}\times 100$$

where *f* is the mass fraction of PLA matrix in the composites, Δ*Hm* is the melting enthalpy of the composites, and $\Delta H_{m}^{0}$ is the melting enthalpy of 100% crystalline PLA (93.7 J/g).

#### 2.4.6. Interfacial Morphology Analysis (SEM)

The specimens of the impact fracture surface were glued by conductive paste on the test table. All specimens were treated with a 10 nm layer of spray gold before observation by an FEI scanning electron microscope (Quanta FEG, Thermo Fisher Scientific Inc., Waltham, MA, USA) with an acceleration voltage of 15 kV.

## 3. Results and Discussion

### 3.1. FTIR Analyses of the Bamboo Fiber

**Figure 1:**
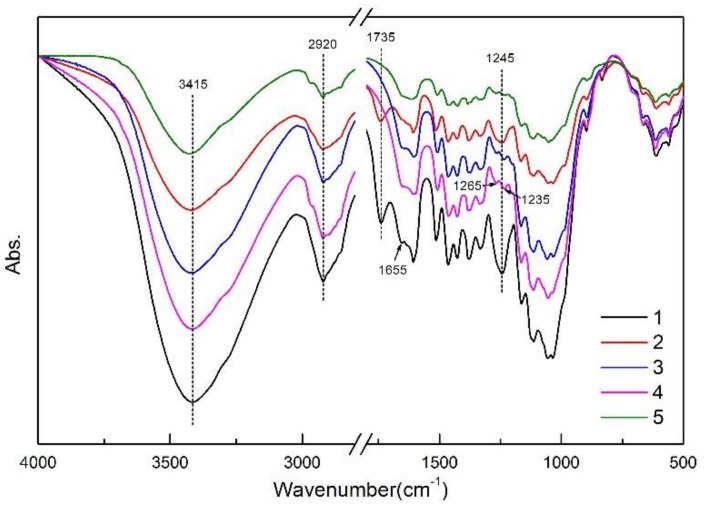
FTIR spectra of treated and untreated BF. (1) UB; (2) DB; (3) ADB4; (4) ADB6; (5) ADB8.

The FTIR spectra of the untreated bamboo fiber (UB), DA-modified bamboo fiber (DB), and alkali/DA co-treated bamboo fiber (4, 6, and 8 wt % of sodium hydroxide treatment named ADB4, ADB6, and ADB8, respectively) are presented in Figure 1.

As can be seen in Figure 1, broad characteristic peaks near 3415 cm^−1^ are designated as the stretching vibrations of amino (–NH) and hydroxyl (–OH) groups. At 2920 cm^−1^, there are wide absorption peaks of the acromion, the symmetric and asymmetric stretching vibration absorption peaks of methyl (–CH3) and methylene (–CH2), belonging to the characteristic absorption peak of cellulose. The characteristic peaks located at 1735 cm^−1^ and 1245 cm^−1^ are assigned to the vibration band of carbonyl (C=O) in hemicellulose and the acyl-oxygen (CO–OR) stretching vibration in hemicellulose and lignin [31]. The five spectra show an obvious difference at 3415 cm^−1^; the intensity of the characteristic peak of the DB here is significantly reduced, indicating that the phenolic hydroxyl and amino groups on the dopamine molecule form hydrogen bonds with the free hydroxyl groups on the surface of the bamboo fiber. The characteristic peak intensity of ADB4 and ADB6 lie between UB and DB, indicating that more cellulose was exposed to alkali treatment, with the surge of the specific surface area and stronger polarity of the bamboo fiber. In the meantime, dopamine treatment failed to achieve dopamine coating under the same conditions, which is why the characteristic peak of ADB6 is stronger than that of ADB4.

The characteristic peak intensity of ADB8 at 3415 cm^−1^ is the lowest, which is attributed to the reaction of alkali and cellulose with the increase in alkali concentration, resulting in the formation of sodium cellulose, which causes the breakage of the surface of the bamboo fiber. The carbonyl (C=O) stretching vibration peaks appeared in UB and DB at 1735 cm^−1^, and the peaks disappeared after alkali treatment. Researchers have reported that certain groups in the hemicellulose will be dissolved by an alkaline solution in the area where the carbonyl absorption peak is located [32]. It can be seen that at 1245 cm^−1^ there is a significant increase in the infrared transmittance of aromatic ether (C–O) after alkali treatment, which also indicates a reduction of hemicellulose and lignin. In summary, alkali treatment can dissolve impurities, wax, hemicellulose, and lignin of bamboo fiber in suitable alkali concentrations, and the appropriate dopamine reaction time can improve the interfacial properties of the composites.

### 3.2. XRD Analyses of the Bamboo Fiber

**Figure 2:**
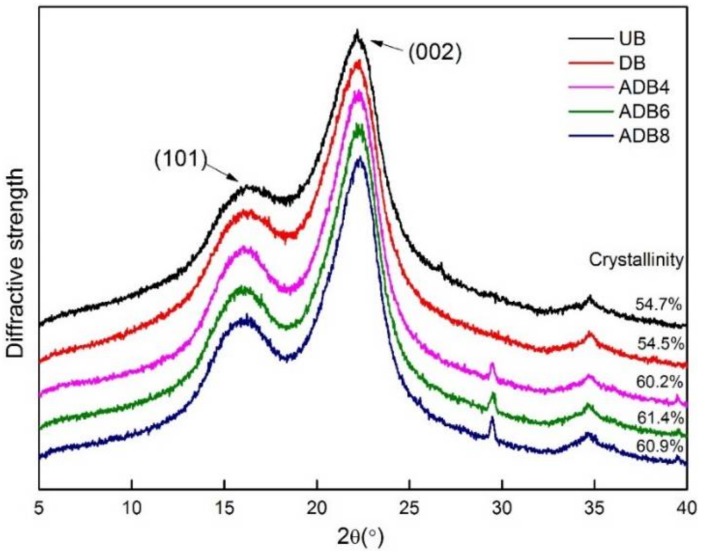
XRD analysis of treated and untreated BF.

Figure 2 exhibits the X-ray diffraction pattern of the treated and untreated bamboo fiber. The two reflections (101) and (002) are used to reflect the crystalline structure in the X-ray diffraction patterns of the natural plant fiber. It can be seen that the characteristic peak of the untreated fiber (101) and (002) correspond to those of the treated bamboo fiber. The positions of the five peaks barely change, and the corresponding diffraction angles are around 16.5° and 22°, indicating that the crystalline region of cellulose is barely affected by the treatment.

The crystallinity of the bamboo fiber barely changed after the modification of dopamine, which could be expected in light of the little effect of the dopamine modification process on the amorphous region, which is also consistent with Hong’s research conclusion [22]. However, the crystallinity of ADB4 increased to 60.2% after alkali treatment. This result could be attributed to the fact that the amorphous region of bamboo fiber was extracted with the dissolution of some hemicellulose and pectin during the alkali treatment, and that the hydroxyl groups of the amorphous microfibrils were then exposed and formed hydrogen bonds with the microfibrils on the surface of the crystalline region. The crystallinity of ADB8 began to decline when the alkali concentration reached a certain level, which is due to the reaction between the crystalline region and the alkali solution, leading to the damage of the crystal structure and partially opening hydrogen bonds. This is under the conclusion of the production of sodium cellulose in the FTIR analysis.

### 3.3. Effect of BF Treatments on Mechanical Properties of BF/PLA Composites

The mechanical properties of bamboo fiber/polylactic acid composites before and after interfacial conditioning treatments are shown in Table 2. Compared with UB/PLA, the flexural strength and tensile strength of DB/PLA composites increased by 13.2% and 22.6%, respectively. This would be due to the high reactivity of dopamine, which can be used as a starting point for graft modification to form chemical links with the matrix materials. In addition, it can serve as a secondary reflection platform to enhance the fiber–matrix interfacial adhesion of the composites, leading to an effective transfer of stress between matrices and fibers. In addition, a significant difference in impact strength was observed between the UB/PLA and DB/PLA composites [17]. For fiber-reinforced composites, the energy propagation in the composites would be hindered with improved interface strength, forming localized stress in the composites. The hindering of the crack propagation through the interface decreased the total energy absorption for composites, thus causing a brittle fracture mode when the composites were loaded [33,34].

After the synergistic treatment, the flexural and tensile strengths of the ADB4/PLA composites were increased by 16.1% and 34.4%, respectively, compared with the UB/PLA composites. The main reason for the above results is that alkali treatment removes some hemicellulose and pectin in the bamboo fiber, which increases the specific surface area of the bamboo fiber and hydroxy groups, resulting in an increase in the physical bonding area between the matrix material and the reinforcement material. At the same time, the PLA melt easily infiltrated into the deep layer of the bamboo fiber to form rubber nails under the action of pressure, which further enhanced the physical combination of PLA and bamboo fiber. However, with the increase in alkali concentration during the synergistic treatment, the flexural and tensile strength decreased to 56.75 and 32.86 MPa when the alkali concentration reached 8 wt %. This could be explained by the fact that sodium hydroxide will de-polymerize and destroy the structure of the cellulose [35,36]. Subsequently, the bamboo fiber will be damaged and its performance will decline. Impact strength increases as alkali concentration increases, even if the trend is not obvious. The main reason for this is that alkali treatment dissolves the impurities and increases the porosity on the surface of the fiber, which can absorb more impact energy and thus improve the impact properties of the composites. Another possibility is that bamboo fiber concentrates the stress at the crack tip and prevents a large region around the crack tip from undergoing either crazing or shear yielding [24]. Based on a comparison with UB/PLA composites, synergistic treatment has effectively enhanced mechanical properties without sacrificing toughness distinctly [37].

### 3.4. The Effect of BF Treatments on the Thermal Properties of BF/PLA Composites

#### 3.4.1. Thermogravimetric Analysis (TG)

**Figure 3:**
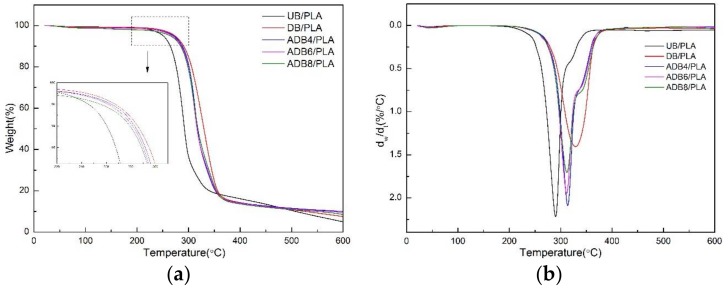
Thermogravimetric analysis (TGA) curves (**a**) and DTG curves (**b**) of various treatments of the effect on thermal properties of BF/PLA composites.

Figure 3 shows the TG and DTG curves based on the PLA matrix composites reinforced by bamboo fiber before and after alkali treatment/DA modification. In addition, the onset pyrolysis temperature (OPT) and maximum rate temperature (MRT) of BF/PLA composites with various treatments are listed in Table 3.

It can be seen in the figure that the composite weight loss is divided into three stages. In the first stage, the DTG curves showed small peaks at about 70 °C, which has been associated with weight loss in moisture evaporation of bamboo fiber. In the second stage, within the range of 230–350 °C, a decline derived from the thermal decomposition of cellulose and hemicellulose is obvious. Both the OPT and the MRT of DB/PLA reached maximum values of 296.33 and 329.32 °C. The UB/PLA shows the sharpest peak of pyrolysis. With the introduction of alkali treatment, the OPT and the MRT began to show a decreasing trend. It can be seen that the thermal stability of DB/PLA is significantly higher than that in UB/PLA, leading to a good coating of dopamine on the surface of the bamboo fiber, which improves the thermal stability of the PLA matrix-based composites. The thermal stability of ADB/PLA is between that of UB/PLA and DB/PLA, because of the dissolution of the lignin, pectin, wax, hemicellulose, and other small molecules in the alkali treatment process, causing many pores in the bamboo fiber system to make the pyrolysis process more conducive to the spread of volatile gases and heat transfer. Therefore, the weight loss rate of ADB/PLA is faster than that of DB/PLA [38]. The third stage is mainly the lignin pyrolysis interval. There is an intersection point between each curve in the temperature range 370–600 °C. DB/PLA and ADB/PLA have a lower rate of weight loss than does UB/PLA between 370 and 480 °C, and reverses between 480 and 600 °C. This is mainly because alkali treatment dissolves some of the lignin, resulting in a lower weight loss rate. Consequently, DB/PLA has the highest thermal stability, and alkali treatment does not improve thermal stability.

#### 3.4.2. Differential Scanning Calorimetry (DSC)

**Figure 4:**
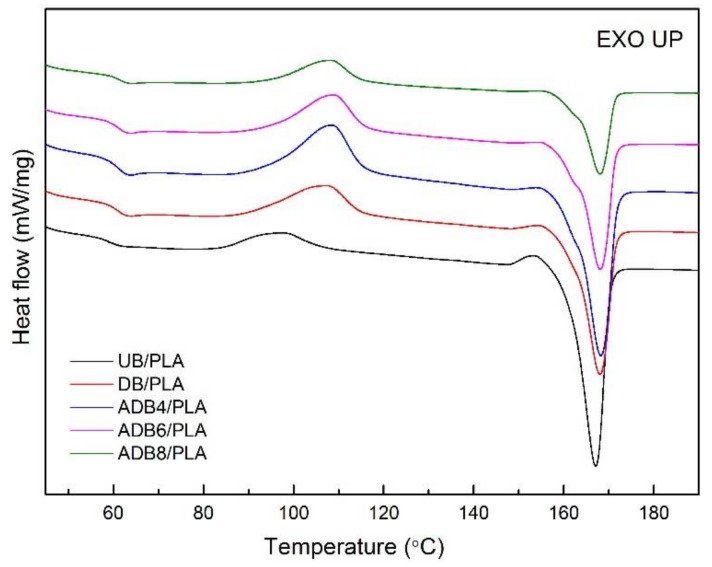
DSC curves of various treatments of effect on thermal properties of BF/PLA composites.

DSC curves and relevant thermal parameters are presented in Figure 4 and Table 4, respectively. Table 4 shows that the *T_g_* of DB/PLA is 2.74 °C higher than that of UB/PLA. This indicates that, after the modification of dopamine, more hydrogen bonds are formed and the molecular chain movement of polylactic acid is hindered [39,40]. However, alkali treatment does not significantly affect the *T_g_* of DA-modified bamboo fiber/PLA composites. It can be seen that the *T_c_* of UB/PLA is 97.42 °C, and the *T_c_* of DB/PLA and ADB4/PLA are 107.32 °C and 108.51 °C, respectively. The change in *T_c_* demonstrates that the treated composites have a strong capability of cold crystallization during the heating process, which may be related to the removal of part of the lignin, hemicellulose, and other impurities in the alkali treatment. This process can play the role of the modified bamboo fiber more effectively as a PLA nucleating agent [29]. In addition, the *T_m_* of UB/PLA is 166.01 °C, and the *T_m_* of DB/PLA and ADB4/PLA is increased to 168.09 and 168.89 °C, respectively, which indicates that the interface performance of these two latter composites is improved compared with that of UB/PLA. Interestingly, the crystallinity of the composites prepared by alkali treatment is lower than that of UB/PLA, probably because the alkali treatment not only removes part of the fiber’s surface material but also destroys the internal structure of the fiber. The reason for the decrease in the crystallinity of DB/PLA is due to the grafting of the dopamine-coated bamboo fiber onto a PLA matrix, which hinders the formation of PLA crystallization [23].

### 3.5. The Effect of BF Treatments on Fracture Surfaces of BF/PLA Composites

**Figure 5:**
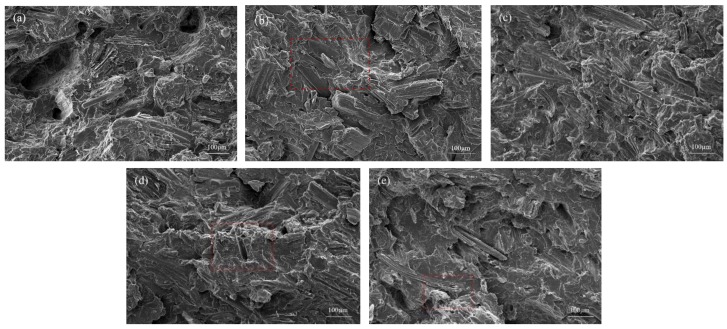
SEM micrographs of the impact cross-section of the BF/PLA: (**a**) UB/PLA; (**b**) DB/PLA; (**c**) ADB4/PLA; (**d**) ADB6/PLA; (**e**) ADB8/PLA.

The surface morphologies of impact cross-section of the composites samples are shown in Figure 5. The influence of different surface treatment methods on the mechanical properties can be explained by analyzing the interface of the composites. As can be seen in Figure 5a, the untreated bamboo fiber surface is relatively smooth, free of voids and defects, and the surface is accompanied by impurities. The fiber bundles with obvious agglomerations on the cross section of the material were pulled out, and there were residual pull-out marks and holes, indicating poor compatibility between the untreated bamboo fibers and the polylactic acid matrix [41,42].

Figure 5b shows the impact cross-section morphology of the composite treated with dopamine. The cross section of the figure is relatively rough, and there are no gaps or holes between the bamboo fiber and the PLA matrix, which indicates that the bamboo fiber is removed and the surface is bonded with the PLA resin. After alkali treatment, the impact cross-section morphology of ADB4/PLA shows no obvious distinction between the two phases. Compared with the cross-section morphology of DB/PLA, alkali treatment separated the fiber bundles and increased the specific surface area of the bamboo fiber. In addition, the image of the surface began to darken, indicating that alkali treatment dissolves low molecular impurities such as pectin, hemicellulose, and lignin, forming a large number of holes, which enhances the physical bond strength between the two phases. With the increase in alkali concentration during alkali treatment, the dopamine-modified surface coating could no longer meet the sudden increase in the cellulose specific surface area under the same conditions. Figure 5d shows that, when the alkali concentration was 8%, the section a tiny hole began to appear, and the impurities between single fibers could be effectively removed. However, the properties after treatment deteriorated, and the bamboo fiber began to irregularly rupture, indicating that the sodium hydroxide destroyed the structure of the bamboo fiber [43,44]. Subsequently, the mechanical properties of the bamboo fiber were not retained, which ultimately resulted in a decrease in the mechanical properties of ADB8/PLA. In summary, the surface modification of the bamboo fiber has an important effect on the properties of the interface between the fiber and the matrix, and appropriate alkali treatment and dopamine modification can effectively improve the overall properties of the composites.

## 4. Conclusions

The introduced dopamine in the composites functioned as a coupling agent to establish chemical connections between bamboo fiber and PLA matrix, which improved the interfacial adhesion of BF/PLA composites and hence their tensile and flexural properties. Further improvements in these mechanical properties were noted when proper alkali-pretreated bamboo fiber was subjected to dopamine modification. The results of XRD show that, after alkali treatment, the non-crystalline region of cellulose was extracted and the crystallinity increased, which is based on the results of FTIR. The TG-DTG curves show that synergistic treatment increases the thermal stability of the composites. In addition, synergistic treatment also controls the thermal properties of bamboo fiber/PLA composites, such as *T_g_*, *T_c_*, *T_m_*, and *X_c_*. The SEM micrographs of impact-fractured specimens also demonstrate that the ADB4/PLA composites have better fiber-matrix adhesion than do the UB/PLA and DB/PLA composites, and this improved adhesion is responsible for the improvement in mechanical and thermal properties.

## Figures and Tables

**Table 1:** The designations of the control and the experimental group in this study. BF: bamboo fiber; DA: dopamine; UB: untreated BF; DB: DA-modified BF; ADB: alkali/DA co-treated BF; PLA: polylactic acid.

Sample Designation		Fiber Treatment Condition	
General	Specific	DA Concentration (wt %)	Alkali Concentration (wt %)
UB/PLA	UB/PLA	No treatment	No treatment
DB/PLA	DB/PLA	1	No treatment
ADB/PLA	ADB4/PLA	1	4
	ADB6/PLA	1	6
	ADB8/PLA	1	8

**Table 2:** Mechanical properties of the fiber-reinforced UB/PLA, DB/PLA, ADB4/PLA, ADB6/PLA, and ADB8/PLA composites.

Sample	Flexural Strength (MPa)	Flexural Modulus (GPa)	Tensile Strength (MPa)	Tensile Modulus (GPa)	Impact Strength (kJ/m^2^)
UB/PLA	55.32(1.98) ^c^	4.29(0.31) ^a^	29.39(2.49) ^c^	1.71(0.08) ^d^	8.13(0.62) ^a^
DB/PLA	62.62(2.99) ^ab^	4.44(0.30) ^a^	37.35(3.15) ^a^	2.07(0.12) ^ab^	6.46(0.59) ^b^
ADB4/PLA	64.25(3.78) ^a^	4.53(0.34) ^a^	39.51(2.61) ^a^	2.17(0.15) ^a^	8.43(0.48) ^a^
ADB6/PLA	58.81(2.16) ^abc^	4.41(0.32) ^a^	38.47(2.36) ^a^	2.03(0.12) ^b^	8.65(0.76) ^a^
ADB8/PLA	56.75(2.77) ^bc^	4.24(0.56) ^a^	32.87(3.03) ^b^	1.87(0.07) ^c^	8.89(1.09) ^a^

Note: The data were analyzed by one-way ANOVA based on a 95% confidence interval. The standard deviations of the test results were recorded in parentheses, the letter markers show the statistical differences. The groups do not differ significantly from one another when they have the same letter(s), and vice versa.

**Table 3:** Thermal parameters of BF/PLA composites with various treatments.

Sample	UB/PLA	DB/PLA	ADB4/PLA	ADB6/PLA	ADB8/PLA
Onset pyrolysis temperature (°C)	270.73	296.33	295.47	293.71	291.58
Maximum rate temperature (°C)	290.09	329.32	313.82	311.62	310.17

**Table 4:** Typical thermal parameters of DSC analysis of various BF/PLA composites.

Sample	*T_g_* (°C)	*T_c_* (°C)	*ΔHc* (J/g)	*T_m_* (°C)	*ΔH_m_* (J/g)	*X_C_* (%)
UB/PLA	58.78	97.42	4.964	166.01	28.86	51.33%
DB/PLA	61.52	107.32	11.28	168.09	24.57	43.70%
ADB4/PLA	60.89	108.51	13.96	168.89	22.36	39.78%
ADB6/PLA	60.93	108.14	13.67	167.76	21.34	37.96%
ADB8/PLA	60.98	108.97	12.83	167.12	20.02	35.61%
